# Leiomyosarcoma, a rare smooth muscle cancer in the pancreas: An adjuvant radiotherapy treatment approach

**DOI:** 10.1002/cnr2.1820

**Published:** 2023-04-24

**Authors:** Ali Taghizadeh Kermani, Alireza Khooei, Mohsen Aliakbarian, Soudeh Arastouei

**Affiliations:** ^1^ Cancer Research Center Mashhad University of Medical Sciences Mashhad Iran; ^2^ Department of Pathology, Faculty of medicine Mashhad University of Medical Sciences Mashhad Iran; ^3^ Mashhad transplant research Center Clinical research institute, Mashhad University of Medical Sciences Mashhad Iran

**Keywords:** adjuvant, leiomyosarcoma, leiomyosarcoma of pancreas, mesenchymal tumor, oncology, pancreas, radiotherapy

## Abstract

**Background:**

Leiomyosarcoma of visceral organs is uncommon, and pancreatic primary occurrence is even rarer. In terms of curative treatment, patients are generally managed with surgery alone, without significant data on the role or efficacy of adjuvant chemotherapy.

**Case presentation:**

This manuscript presents a case of a 22‐year‐old female with advanced primary leiomyosarcoma of the pancreas, treated with radical surgery and adjuvant radiation therapy.

**Conclusion:**

With a low‐survival rate, consideration of radiation therapy in some advanced and unresectable cases could be potentially beneficial.

## 1 INTRODUCTION

Leiomyosarcoma of visceral organs is uncommon, rather it is most commonly encountered in the gastrointestinal tract or the uterus. Primary leiomyosarcoma of the pancreas (PLMS) is a rare event, accounting for two of every 5075 cases of leiomyosarcomas,^1^ and one in every 7129 pancreatic tumors in general.^2^

Due to the rarity of the disease and the lack of evidence for best practices, its management is controversial. A comprehensive search revealed reports of the disease that mainly focused on clinicopathologic and imaging features, and revealed that many patients were managed with surgery alone. This case report presents a young female patient with PLMS, invading the peripancreatic tissue, who was treated with radical surgery and adjuvant radiation therapy,

## 2 CASE PRESENTATION

A 22‐year‐old female presented to a gastroenterologist in Mashhad, Iran in March 2020, with the chief complaint of abdominal pain lasting for more than 1 year. The pain had progressively worsened and she had developed icterus a week prior to her presentation. She did not have any loss of weight, and no other comorbidities.

**FIGURE 1:**
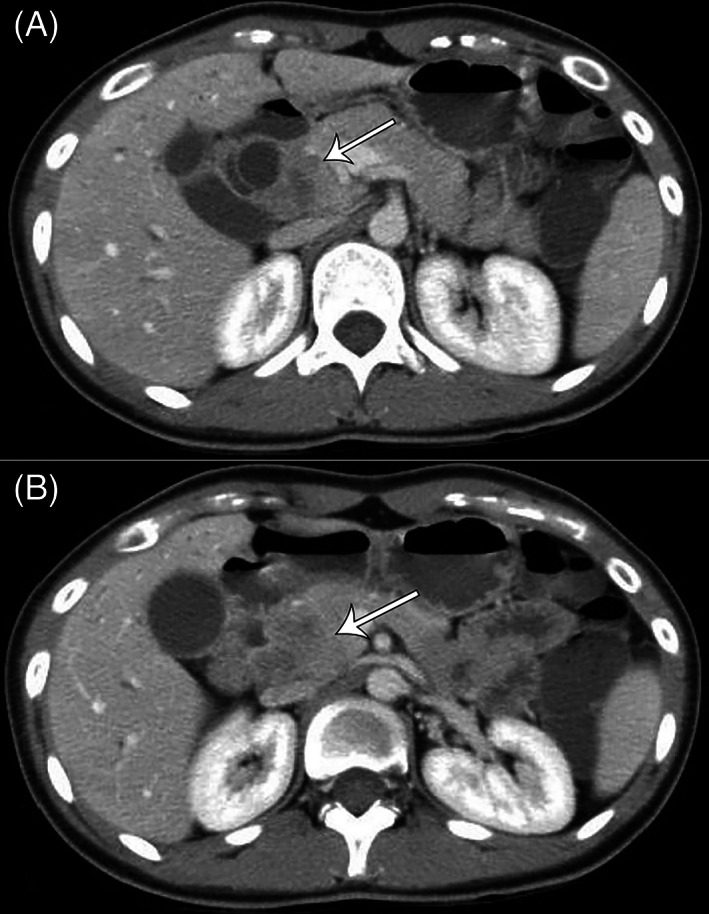
Spiral Computerized Tomography (CT) scan of the abdomen with intravascular (IV) and oral contrast (A, B) showcases a bulged head of pancreas with heterogeneous enhancement, hypodense foci measuring 15× 30 mm is seen in the corresponding area, in close proximity to hepatic vessels. Gallbladder, cystic and common bile ducts are dilated.

The initial abdominal ultrasound revealed dilation of bile ducts. Computed tomography (CT) scan illustrated a hypodense mass in the head of pancreas, suspected to be a focal pancreatitis or a pancreatic mass (Figure 1). Magnetic resonance cholangiopancreatography (MRCP) revealed a lobulated pancreatic mass measuring 2 × 2.2 cm in size. The patient was referred for surgery, and during surgery, a tumor originating from the pancreatic head, located close to the hepatic artery, was revealed. A Whipple resection with extended lymphadenectomy was performed. The postoperative period was uneventful and she was discharged after 1 week.

**FIGURE 2:**
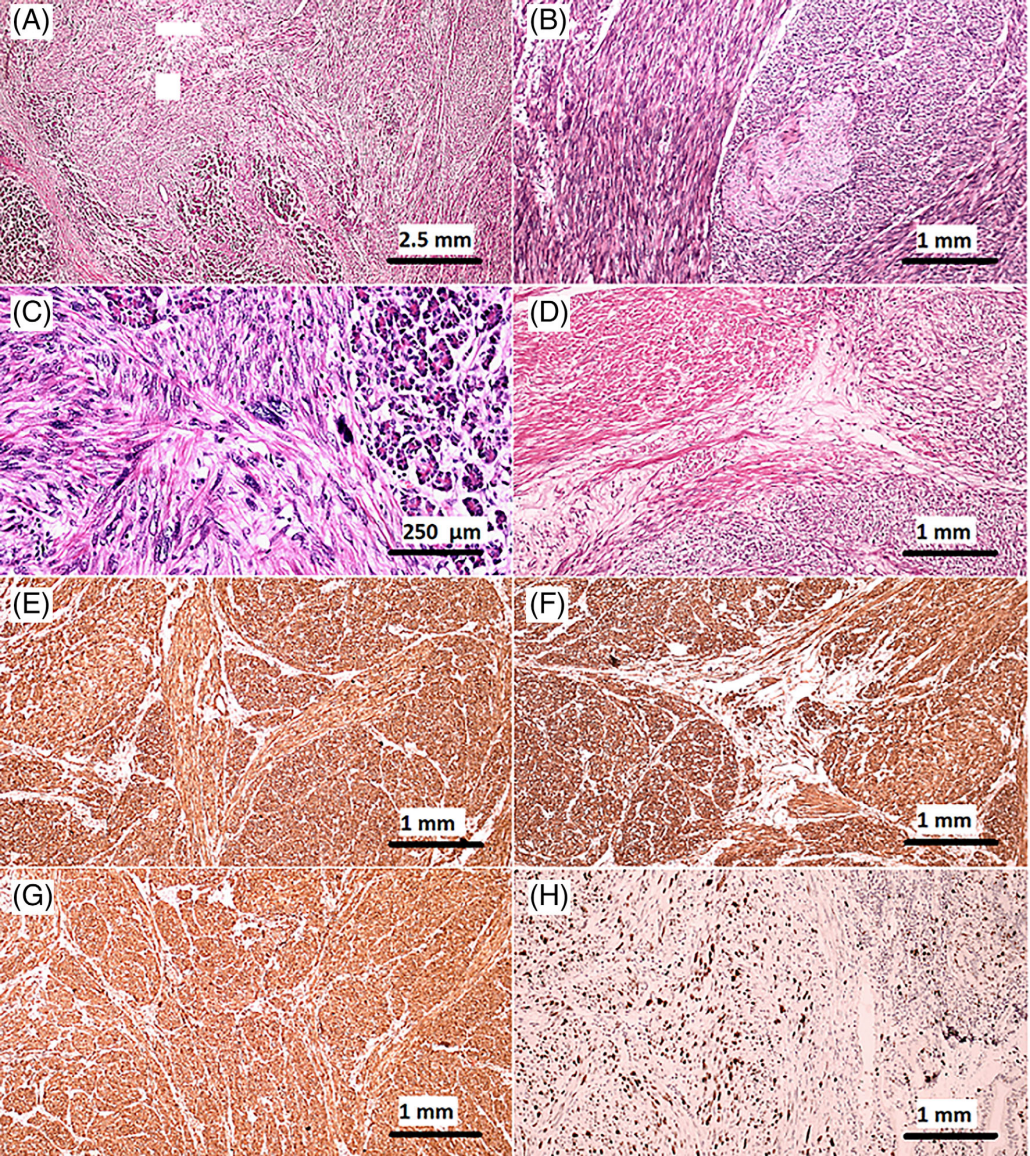
Histologic features of tumor tissue. (A–D) Hematoxylin and eosin staining: (A) (40× magnification) represents a fascicular pattern of proliferating spindle cells invading pancreatic exocrine secretory acini. (B) (100× magnification) shows entrapping of a neural bundle by tumoral cells. (C) (400× magnification) shows spindle shaped blunt ended nuclei of tumoral cells, depicting obvious cellular atypia. (D) (100× magnification) represents focal obvious smooth muscle differentiation of tumoral cells, represented by deep eosinophilic staining of their cytoplasm. (E–H) Positive immunohistochemical markers of tumor tissue: (E) shows extensive positive immunoreactivity of tumoral cells for smooth muscle Actin (SMA), (F) showcases another extensive positive immunoreactivity of tumoral cells for Desmin, (G) shows Extensive positive immunoreactivity of tumoral cells for Caldesmon (H) represents high‐proliferating index, as demonstrated by strong nuclear positive immunoreactivity of tumoral cells for Ki67 marker, despite infrequent mitotic figures in H&E stained slides.

Gross pathological examination demonstrated a firm, creamy, infiltrating tumor mass, measuring 4.5 × 4 × 4 cm, located in the pancreatic head, invading the peripancreatic tissue and an adjacent lymph node. The tumor had focally invaded the duodenal wall, but did not seem to be originating from it. Microscopic and immunohistochemistry studies identified the tumor as leiomyosarcoma of the pancreas (Figure 2A–H).

By May 2020, 1 month postsurgery, she was completely active with an Eastern Cooperative Oncology Group (ECOG) Performance Status score of 0, and free of symptoms. On physical examination neither organomegaly nor tenderness of the abdomen were detected. After presenting the case to a multidisciplinary care team, and discussing the benefits and drawbacks of all available adjuvant options, she was then further treated with adjuvant external beam radiotherapy at a dose of 5040 cGy in 28 fractions by intensity modulated radiotherapy technique on the basis of invasion to neighboring organs.

After 20 months of follow‐up, she has been disease free as evidenced by chest and abdominopelvic scans obtained every 3 months. She is completely active with an ECOG Performance Status of one. She only complains of abdominal discomfort, weight loss, digestive problems, and chronic fatigue.

## 3 DISCUSSION

Pancreatic leiomyosarcoma is a rare mesenchymal tumor accounting for less than 0.1% of all malignant pancreatic (non‐islet) cancers. This malignancy has a uniquely aggressive behavior that affects both adjacent and distant organs. The rarity of this disease and the lack of research and available data on most effective treatments, particularly in young patients, demonstrates the need for this case report and others like it.

First described by Ross in 1951, thereafter, cases of pancreatic leiomyosarcoma have primarily been addressed through case reports and managed by radical resection; however, a multidisciplinary team decided to treat our case with adjuvant radiotherapy due to focal invasion of neighboring organ. A recent review of clinicopathological features of 88 patients with primary leiomyosarcoma of the pancreas showed that most cases occurred during the fifth decade of life with the youngest being 14 years old.^3^ Patients commonly presented with abdominal pain and mass, 30% with local invasion to adjacent organs, and another 30% with stage IV disease. Roughly, 60% of those studied were candidates for radical curative surgery.^3^ Our patient was on the younger end of this spectrum, presented with similar symptoms, and showed invasion into the adjacent duodenal wall and peripancreatic fat.

While patient age, tumor size, vascular invasion, and mitotic count have generally been proposed as important prognostic factors for leiomyosarcoma, a systematic review demonstrated slightly different results in leiomyosarcomas of pancreas: nonradical resection was an independent adverse prognostic factor, invasion to adjacent organs/vessels was a potential adverse factor, while size and age showed no significant effect.^4^ Another analysis found radical resection to be the sole prognostic factor in nonmetastatic cases, and reported invasion into neighboring organs to be insignificant.^3^

However, the most important prognostic factor agreed upon in the literature is radical surgery.^3^, ^4^, ^5^ Our patient also underwent radical surgery with negative surgical margins. After complete surgical resection, local recurrence is mainly seen in cases with invasion to adjacent organs. A patient with a 5.5 cm pancreatic body leiomyosarcoma invading the adjacent intestinal tract was observed following radical surgery, and returned with local recurrence 15 months after radical surgery.^6^ Another patient with an 8 cm mass in the body and tail of pancreas invading the mesocolon experienced local recurrence within 5 months after radical surgery.^7^

However, some cases with local invasion to adjacent organs with durable disease‐free survival have also been reported. For example, a case of PLMSP in the head of the pancreas with invasion into the ampullary region and common bile duct, was disease free 19 months postsurgery, without any adjuvant treatment.^8^ Another case with a similar tumor invading the duodenal wall, was also disease free for a year with a similar therapeutic strategy.^9^

Data on the use of adjuvant treatment in this disease is extremely lacking. To the best of our knowledge, only 3 reported cases underwent adjuvant treatment (Table 1). A 40‐year‐old male with pancreatic tail and body mass invading the spleen and transverse colon, was treated with adjuvant chemotherapy after radical resection, but developed liver metastases 3 months after surgery while on adjuvant treatment.^10^ The other two cases underwent adjuvant radiotherapy; one had an event free survival of 11 months and the other for at least 27 months.^2^, ^11^ Our patient, also treated with adjuvant radiotherapy due to local invasion of duodenum, has been disease free for the past 20 months.

**TABLE 1:** Effect of adjuvant treatment on survival in published cases reports

Study	Year	Age	Sex	Location in pancreas	Size (cm)	Local invasion	Treatment	DFS (at least)
Kim et al.^2^	2014	51	F	Tail	5.5	NA	DP^a^/RT^b^	27 months
Lalim et al.^10^	2012	40	M	Tail and body	20	spleen and transverse colon	DP/CT^c^	3 months
Omidvari et al.^11^	2016	56	M	Tail	12	No	DP+ splenectomy/RT	11 months

^a^ DP = distal pancreatectomy.

^b^ RT = radiation therapy.

^c^ CT = chemotherapy.

The national comprehensive cancer network guidelines recommend consideration of pelvic EBRT in completely resected stage II‐III (i.e., extrauterine, nonmetastatic disease) uterine leiomyosarcoma.^12^ Therefore, extrapolating these results to pancreatic leiomyosarcoma seems appropriate, as there is paucity of data regarding the adjuvant treatment in this tumor site.

The effectiveness of RT is further demonstrated in a report of an unresectable, nonmetastatic PLMSP patient who refused chemotherapy and underwent palliative radiotherapy instead. He received 54Gy over 30 fractions and was clinically well for 18 months.^13^

## 4 CONCLUSION

The primary leiomyosarcoma of the pancreas is an extremely rare entity and data about its diagnostic consideration and management is grossly insufficient. However, due to high mortality rates, adjuvant treatments following a Whipple procedure is crucial. While requiring longer follow‐ups, there is cautious optimism about the potential benefits of radiation therapy in the management of this rare malignancy.

## AUTHOR CONTRIBUTIONS

The authors listed are the sole authors of this manuscript. **Ali Taghizadeh Kermani**: conceptualization (equal); supervision (lead); resources (equal); writing – review and editing (equal). **Alireza Khooei**: Data curation (equal); resources (equal); writing – original draft (supporting); writing – review and editing (supporting). **Mohsen Aliakbarian**: data curation (equal); resources (equal); supervision (supporting); writing – review and editing (supporting). **Soudeh Arastouei**: data curation (equal); writing – original draft (lead); conceptualization (lead); Writing – review and editing (equal).

## FUNDING INFORMATION

The authors received no specific funding for this work.

## CONFLICT OF INTEREST STATEMENT

The author declares there is no potential conflict of interest.

## ETHICS STATEMENT

A written informed consent was obtained from the patient for this manuscript.

## Data Availability

The data that support the findings of this study are available from the corresponding author upon request
